# Pharmacist Led Intervention on Inhalation Technique among Asthmatic Patients for Improving Quality of Life in a Private Hospital of Nepal

**DOI:** 10.1155/2019/8217901

**Published:** 2019-11-24

**Authors:** Anita Yadav, Parbati Thapa

**Affiliations:** Pharmaceutical Sciences Program, School of Health and Allied Sciences, Pokhara University, Pokhara-30, Kaski, ZIP/Post code 33700, Nepal

## Abstract

**Purpose:**

Asthma is a chronic disease which cannot be cured but can be controlled. Although drug therapy is used to relieve and prevent symptoms of asthma and treat exacerbations, still a good asthma control and a better quality of life in many patients is suboptimal due to improper use of inhalation technique. Thus, this interventional study was conducted to evaluate the effect of a pharmacist intervention on asthma control, quality of life and inhaler technique in adult asthmatic patients.

**Patients and Methods:**

A total of 72 patients who met the inclusion criteria and agreed to give written consent were enrolled in the study. These patients were randomly divided into two groups i.e., test group (36) and control group (36) by simple block randomization technique. Test group were the interventional groups. Mini Asthma Quality of Life Questionnaire (AQLQ), Asthma Control Questionnaire (ACQ) and structured questionnaires were used to sort the information like quality of life, asthma control and demographic details. They were counselled by the pharmacist about the asthma management and proper use of inhalers. Out of 72 patients, only forty six patients came for follow up after one month. Data were entered and analyzed using Statistical Package for Social Sciences (SPSS) software version 20.

**Results:**

A significant change was observed in the mean score of quality of life (*p* = 0.001) in test group as well as control group, however change in the mean score of asthma control in the test group (*p* = 0.001) was more significant as compared to the control group (*p* = 0.099). Inhalation technique was found to be improved significantly after intervention among patients using the metered dose inhaler and dry powder inhaler. Majority of the patients were prescribed with Methylxanthines (24.5%) followed by combined Beta 2 agonists and Inhaled Corticosteroids (21.7%).

**Conclusion:**

Pharmacist provided intervention improves the quality of life, asthma control and inhalation technique among asthmatic patients.

## 1. Introduction

Asthma is a disorder marked by varying interruption of the expiratory airflow arising from acute airway infection that generates distinctive signs of wheeze, breathlessness, tightness of the chest and cough that differ with time and intensity [1]. Poor inhalation is a significant problem in asthma because the individual does not receive an adequate dose of the prescribed drug that leads to reduced therapy and control of asthma [2]. Video aided programs are more effective on providing counselling about the correct inhalation technique than through traditional patient education techniques of reading or lecturing. Each sort of inhaler has distinct methods so, if it is not correctly advised to the patients, they will not follow correct technique which leads to less possibility of illness healing. Thus, it is utmost necessary to counsel the patients about right inhaler technique to optimize their illness [3]. The significant benefit of correct inhalation technique is that medication is supplied to the lungs and that local concentrations of inhaled drugs are higher and systemic side effects are considerably less likely. Using an inhaler device includes a complicated sequence of measures that need to be done properly. Failure to execute one or more measures properly can significantly decrease the delivery and hence the efficacy of the drug. It also contributes to uncontrolled state of disease, unwanted side effects and may also result in greater therapy expenses [4]. There are nearly 1.5–2 million asthmatic patients in Nepal. A large percentage of the population in Nepal is illiterate; therefore, the need for adequate inhalation technique is even more essential. Thus, it is necessary to assess the knowledge of the patients regarding the use of inhalers and then training should be provided for the correct and proper use of their inhaler. Pre-post interventional study conducted by Manandhar et al. in Nepal found that the inhalation technique of asthma patients was found to be poor; only 45 out of 160 patients were able to perform all the steps correctly before intervention as compared to 147 patients after single intervention [5]. The studies revealed that the level of asthma control and quality of life in the intervention group was increased more than the control group (*p* = 0.039) at 0.05 level of significance after the pharmacist provided intervention. Pharmacist-led interventions in asthma patients not only enhance the inhaler technique but also enhance medication adherence, asthma control and quality of life [6–8]. Pharmacists provided intervention on inhalation technique results in the control of asthma as evaluated by the Asthma Control Test (ACT). The intervention decreased the use of reliever drug and the incidence of night-time awakenings due to asthma. Adoption of correct inhalation technique was considerably greater in the intervention group [9]. The National Asthma Education and Prevention Program 1989 outlined the role of pharmacist in defining issues linked to uncontrolled disease state, educating patients about their medicines and monitoring them. Finally, it concluded that pharmacist education and supporting role can contribute to improve asthma control and enable patients to live full active and productive lives [10]. Uncontrolled asthma has a major effect on the quality of life of the patients. The extent of morbidity is influenced by several variables such as age, financial status and asthma-related co-morbidities [11]. Although, many studies had been conducted in Nepal, regarding the inhalation technique but the study related to asthma control and quality of life is limited. In patients with chronic disorders, which are not curable but only treatable, quality-of-life assessment may be even more relevant, as achievement of the best possible quality of life becomes the major goal of patient management. Our study aimed to evaluate the effect of a pharmacist intervention on asthma control, quality of life and inhaler technique in adult patients suffering from asthma.

## 2. Materials and Methods

### 2.1. Research Design

The pre and post interventional study was conducted to evaluate the effect of a pharmacist intervention (intervention provided by registered pharmacist who is the principle investigator) on asthma control, quality of life and inhaler technique in adult patients suffering from asthma. Patients were divided randomly into the test and control group by simple block randomization technique. Patients were followed up after one month and the pre intervention data and post intervention data were compared to evaluate the effect of pharmacist intervention on reducing errors on the use of inhalers, improving quality of life and measuring asthma control towards asthma therapy. Dependent variables were asthma control, quality of life and inhalation technique. Independent variables were age, gender, marital status, family type, education status, family source of income, and duration of asthma. Study was conducted at Medicine OPD (Out Patient Department) of Crimson Hospital, which is situated at Tilottama-05, Manigram, Rupandehi, Nepal. The study was carried for period of six months from June to November 2018. Census sampling technique was applied for the data collection so the entire patients diagnosed with asthma and meeting the inclusion criteria during the data collection period of August to November 2018 were included in the study.

### 2.2. Study Population

The patients aged ≥18 years of age of either gender, clinically diagnosed with asthma with or without co morbidities and who were on inhalers and/or medications for their asthma were included in the study. Asthmatic patients admitted in emergency department and who were not willing to give consent to participate were excluded.

### 2.3. Ethics Approval

Ethical approval for the research was obtained from concerned authority that is Institutional Review Committee of Pokhara University with reference number (Ref. No. 17-075-76) and Helsinki Ethical Guidelines was followed. Prior permission to conduct the study was obtained from Crimson Hospital, Tilottama-05, Manigram, Rupandehi, Lumbini Zone, Nepal with reference number 532-074-075. Written informed consent was obtained from the patient before enrolling them in the study. Patients were fully informed about the purpose and nature of study in the local languages i.e., Bhojpuri and Hindi. Personal details of the patients were kept confidential and anonymity was maintained.

### 2.4. Data Collection Tools

#### 2.4.1. Structured Questionnaire for Demographic Details

Structured questionnaire was designed to collect demographic information [6].

#### 2.4.2. Mini Asthma Quality of Life Questionnaire (MiniAQLQ) and Asthma Control Questionnaire (ACQ)

MiniAQLQ and ACQ were requested through email and hard copy of this questionnaire was obtained via airmail from Professor Elizabeth Juniper, Faculty, Department of Clinical Epidemiology, McMaster University, Canada. Both are self administered or interview based questionnaire used for measuring the quality of life in asthma patients. MiniAQLQ consists of fifteen questions with seven optional answers in which “1” equal to severe impairment and “7” equal to no impairment. The mean score of 4 indicates moderate, <4 indicates poor and >4 excellent quality of life. ACQ consists of seven questions and six responses in which one equal to totally controlled and six equal to extremely poorly controlled. The mean score of ≥1.5 indicates uncontrolled asthma and <1.5 indicates controlled asthma [12].

The patients were interviewed based on Hindi version of miniAQLQ and ACQ. As most of the people speak and understand Bhojpuri language similar to Hindi language, validated Hindi version of questionnaire provided by the developer was used.

#### 2.4.3. Checklists for Metered Dose Inhaler (MDI) and Dry Powder Inhaler (DPI)

The checklists adapted from Sodhi et al. for MDI and DPI are an excellent tool to assess the correct use of these devices by patients and health care professionals [13]. MDI consists of total eight steps and DPI consists of eleven steps in which correct steps performed were given score one and incorrect steps performed were given score zero. These checklists were used to assess the inhalation practice of the patients immediately after completing the administration of questionnaires to the patients in the test group only. These checklists were again reassessed after one month follow up.

#### 2.4.4. Feedback Form for Verbal and Video Aided Method and Drug Treatment Chart

The feedback form for measuring effectiveness of patient counseling in physical/verbal and video demonstration of inhalation technique and drug treatment chart were prepared by following various published studies [14] and also by consulting research supervisor and was further approved from the consultant physicians who were involved in the treatment of asthma at the study site. The drug treatment chart was used for recording drugs prescribed in the treatment of asthma and leaflet was designed to give complete education about asthma management.

### 2.5. Intervention Tool

Leaflet was the intervention tool used in the study. Patients were provided the counselling with the aid of the leaflet. Leaflet was prepared using the standard reference and was further approved from the supervisor and physician at the Respiratory Unit of hospital.

### 2.6. Validity and Reliability of the Study Tools

The Mini Asthma Quality of Life Questionnaire is one of the short versions of Asthma Quality of Life Questionnaire which has been fully validated by Professor Elizabeth Juniper, Faculty, Department of Clinical Epidemiology and Biostatistics, McMaster University, Canada for use in both clinical practice and clinical trials. It has good reliability and cross-sectional validity. All questionnaires also have strong responsiveness and longitudinal validity [15]. The Asthma Control Questionnaire has been fully validated and can identify the level of control experienced by individual patients and it can pick up small changes that occur as a result of intervention [16]. The feedback form and drug treatment chart were approved by the physicians involved in providing treatment.

### 2.7. Data Analysis

All the data were entered and analyzed using SPSS (Software Package for Statistical Sciences) version 20 and MS-Excel version 2007 for statistical analysis. Descriptive indexes including frequency, percentage, mean and standard deviations were used to express data for all variables. Associations between the variables were analyzed by using paired sample *t* test. A *p*-value of <0.05 was considered as statistically significant throughout the study. Finally the data were represented as tables and figures as appropriate.

### 2.8. Baseline Assessment of Inhaler Techniques and Intervention

After patient selection based on inclusion and exclusion criteria, patients were divided as test and control group. Patients with serial number odd were grouped as test and even number as control. Then questionnaires were filled through face to face interview. Test group patients were provided with intervention however control group were provided the intervention at the end of the study period. The intervention was carried out outside the Medicine-OPD and patients were counselled for nearly 20–25 min. The video aided materials were shown to the patients at their follow up period at the same place.

## 3. Results

A total of 72 patients were enrolled in the study and were divided as test and control, 36 in each group. However forty six patients could be met up after follow up, 25 in test group and 21 in control group.

### 3.1. Baseline Demographic Patterns of Patients

The demographic details of the patients are summarized in Table 1. Majority of the participants were in the age group of >59 years, 29 (40.3%). Genders wise analysis showed that females were predominant over males as females were 40 (55.6%) and males were 32 (44.4%). Among the study population, 29 (40.3%) were illiterate and 21 (29.1%) patients mentioned remittance as family source of income. The study further revealed that 35 (48.6%) of the study population in both the groups had asthma for more than two years.

### 3.2. Assessment of Quality of Life of Asthmatic Patients before and after Intervention

Quality of life of asthmatic patient was assessed using the questionnaire (MiniAQLQ). Data were analysed using paired sample *t*-test which showed a significant increase in mean scores from the baseline (before intervention) to after intervention (*p* = 0.001) in both the groups. The time gap between before and after intervention was one month.

Similarly mean score was compared between the test and control group before as well as after intervention using one sample *t*-test which showed a significant difference in the QOL (Quality Of Life) score between the groups. Details of the analysis have been shown in Table 2.

Table 3 shows the quality of life categorization based on the score. Twenty two patients had poor quality of life and three patients had excellent quality of life before intervention in the test group which got improved after intervention, all 25 patients had excellent quality of life. In the control group also the result showed that quality of life has been improved.

### 3.3. Assessment of Asthma Control of Asthmatic Patients before and after Intervention

ACQ (Asthma Control Questionnaire) was used to assess whether asthma is in control or not and paired sample *t*-test was used for analysis. There was a decrease in mean scores from the baseline (before intervention) to the last visit i.e., after intervention (*p* = 0.001) in test group. Decrease in score signifies the control over asthma as shown in Table 4. However in control group though there is slight decrease in score it was not statistically significant (*p* = 0.099).

Similarly mean score was compared between the test and control group before as well as after intervention using one sample *t*-test which showed a significant difference in the level of asthma control score between the groups. Details of the analysis have been shown in Table 4.

All the patients in the test group had uncontrolled asthma which got improved after intervention, only two patients had uncontrolled asthma even after intervention. However, in control group only four patients had controlled asthma at the end of the study as shown in Table 5.

### 3.4. Assessment of Inhalation Technique before and after Intervention

Out of 36 patients in the test group, 11 patients lost follow up therefore 25 patients remained in the test group. All these 25 patients were using inhalers, among them 16 patients were using Metered Dose Inhaler (MDI) and 9 patients were using Dry Powder Inhaler (DPI). Intervention regarding the correct use of either MDI or DPI was delivered to the patient using the leaflet and counselling. Paired sample *t*-test was used to observe if there occurs any significant change in inhalation technique score before and after intervention.

### 3.5. Metered Dose Inhaler Technique Score Chart

The Table 6 showed the mean scores of the steps while performing MDI technique. The mean score before intervention was 5.68 ± 1.35 whereas the mean score after intervention was 7.25 ± 0.57. There was a significant difference in the inhalation technique score before and after intervention (*p* = 0.001).

Among the steps of MDI using technique, the most incorrectly performed step during the first visit (before intervention) was 7^th^, followed by 8^th^and 6^th^ steps respectively which are shown in Table 7.

### 3.6. Dry Powder Inhaler Technique Score Chart

The mean score of DPI technique before intervention was 8.55 ± 0.88 whereas the mean score after intervention was 10.44 ± 0.72. There was a significant difference in the inhalation technique score before intervention and after intervention (*p* = 0.001) as summarized in Table 8.

Among the steps of DPI using technique, the most incorrectly performed step during the first visit (before intervention) was 5^th^, followed by 8^th^and 9^th^ steps respectively which are shown in Table 9.

### 3.7. Measurement of Effectiveness of Various Types of Demonstration in Inhalers

To evaluate the effectiveness of demonstration (verbal and video aided) technique patients in test group were requested to fill up the feedback form containing four questions soon after providing verbal counselling at first visit. After 1 month during the follow up time reassessment was done on the inhalation technique. At their last visit, video aided counselling was again provided and were requested to fill up the feedback form on the video aided method as well. The measurement of effectiveness of various type of demonstration in inhalers is shown in Table 10.

### 3.8. Medication Management in Asthmatic Patients

The total number of prescriptions was 72 and total number of drugs prescribed was 179. Hence the average number of drugs per prescription was 2.4. Methylxanthines was prescribed to most of the patients i.e., 44 (24.5%) followed by beta 2 agonists (Inhaled as well as oral) and inhaled corticosteroids in combined therapy i.e., 39 (21.7%). Details of the prescribed drugs are shown in Table 11.

## 4. Discussion

The current study showed that there was a significant increase in the mean score of quality of life of asthmatic patients in both the groups i.e., test group and control group (*p* = 0.001). There was no separate place for counselling so it was conducted at open place outside Out Patient Department, so the patients in the control group might have been aware of it and have been influenced which result in significant change in QOL score among control group as well. In Nepal, communication between a people on matters related to health and social issues is common. So, there might be the sharing of information between the people in the test group with control group as well. However, study conducted by Bender et al. [17]; Gamble et al. [18] showed no significant change in quality of life of asthmatic patients in pharmaceutical care group compared to usual group after intervention. The increased QOL score after intervention in test group might be due to the counselling provided by the pharmacist using leaflets, physical and verbal as well as video aided counselling on the use of inhalers.

In this study, the mean ACQ score before intervention and after intervention has been decreased from 3.54 to 1.15 (*p* = 0.001) in the test group and 3.19 to 2.65 (*p* = 0.099) in the control group, similar finding was found in Giraud et al. [19] where mean ACQ score decreased from 1.8 to 1.4 (*p* = 0.001). The significant change in the ACQ score before and after intervention in the test group might be due to the intervention provided by the pharmacist.

A study conducted in Spain by Garcia-Cardenas et al. [20] in the intervention group mean ACQ scores significantly improved (*p* = 0.001) and the number of patients with controlled asthma increased by 30.1% (*p* = 0.001) after six months. The intervention also resulted in improved medication adherence and inhaler technique (*p* = 0.001). No significant changes for any of these variables were observed in the control group. These findings were similar to this study.

Similar type of result was observed in study by Mehuys et al. [9] i.e., mean ACT scores did not change from baseline for both study groups. However, a pre-defined subgroup analysis of patients having insufficiently controlled asthma at baseline showed that the intervention had significantly increased the ACT score after 6 months compared with usual care (*p* = 0.038). The intervention also reduced, for the complete study group, reliever medication use (*p* = 0.012) and the frequency of night-time awakenings due to asthma (*p* = 0.044). Inhalation technique (*p* = 0.004) and adherence to controller medication were significantly better in the intervention group (*p* = 0.016).

A study conducted in Iran by Zaraei et al. [21] showed a statistically significant difference between the amounts of change in asthma control of the two groups, whereas a worsening asthma control was observed in the control group. The changes showed that the intervention was effective in the improvement of asthma control which is similar to this study.

Among 25 patients in test group, 16 (64%) patients were found to use MDIs and 9 (34%) patients used DPIs. This finding is in concordance with the finding of Ocakli et al. [22] which also stated that most selected form of inhaler therapy was metered dose inhaler than dry powder inhaler. The reason behind more users of MDI as compared to DPI might be it is cheaper and convenient to use than DPI in developing countries like Nepal.

Regarding inhalation techniques, the most common errors patients made while using MDI and rotahaler were “not exhaling to residual volume” and “not holding breath for 10 s” which is similar to the study conducted by Palen et al. [23]. However, van Beerendonk et al. [24] found that “not continuing to inhale slowly after actuation of the inhaler” and “exhaling before the inhalation” to be the most common errors.

In this study, the most frequently performed error in inhalation technique was in step 7 (hold breath for 10 s or if possible) and step 8 (Wait for at least one minute before taking the second dose). This finding is in agreement with another study done by Sodhi [13] in India. The majority of the patients were unable to hold breath for 10 s may be because of decreased pulmonary function due to asthma. Secondly, the most common error performed was step 8, because patient might be unaware about the importance of pause between “puffs,” which is required for proper delivery of drugs.

In case of dry powder inhaler the most commonly mistaken step was “Breathe out gently to residual volume away from the mouth piece” which is similar to findings of studies by Palen et al. [23]; Pun et al. [25] and Sapkota and Amatya [26] where 66, 84, and 61% participants respectively made error in exhaling to residual volume. When mistakes are done during the inhalation procedure, significant amount of drugs may fail to reach the lungs. Exhale (breathing out) gently to residual volume is an important step, as without an adequate exhalation, patients may be unable to inhale forcefully and deeply enough through their inhaler in order to ensure deposition of drug into the lungs. Breathing out should be done away from the device. Breathing out into or through DPI may blow away the drug or form lumps of drug inside it making the drug less effective.

In the present study, there was statistically significant improvement in MDI technique (mean score increased from 5.68 to 7.25, *p* = 0.001) and DPI technique (mean score increased from 8.55 to 10.44, *p* = 0.001) after intervention which is similar to the studies done by Basheti et al. [27]; Pothirat et al. [28], which showed that pharmacist counselling on inhalation technique is important to enhance the knowledge in correct use of inhaler devices. Study conducted by Gracia Antequera et al. [29] concluded that patients receiving instruction of inhaler device use, more than once over a period of time showed improvement in their handling of inhaler devices. This result was in agreement with several other studies which showed positive correlation to inhalation technique with counselling (Hammerlein et al. [4]; Lavorini et. al. [30]). These results suggested that repeated training and demonstration can improve the inhalation technique in patients. So, patients using inhaler devices get benefit from regular assessment of their technique and feedback, which ensure compliance and adequate delivery of drug.

As our study sampled more old patients (>59 years), these age group preferred to understand the verbal method rather than video method of demonstration therefore in this study, majority of the patients responded equally to the verbal method and video method.

The proportion of patients following good technique was significantly more in the video group than the oral instruction group at immediate assessment and also at one month post intervention (*p* = 0.0262 and *p* = 0.0075 respectively) by the study conducted by Arumugom et al. [31] which is in contrast to our study. This might be because practical demonstrations received by patients lead to better inhaler technique outcomes than oral or printed instructions received by patients.

In this study, methylxanthines was prescribed to majority of asthmatic patients. This may be due to methyxanthines are widely available in lower costs. They also have anti inflammatory effect on airways. This finding is in agreement with study carried out by Shimpi [32]. This result is in contrary to study done by Arumugam et al. [31] and Kumar et al. [33] where methyxanthine class of antiasthmatic drugs (80%) and (40%) was found to be among the maximum used category.

## 5. Conclusions

The findings of this study showed that there was an effective improvement in the quality of life in both the groups (test and control) however, asthma control status of test group in comparison to the control group was better. The pharmacist led intervention with the help of leaflet, physical and verbal as well as video demonstration of inhalation technique was proved to be effective in the improvement of QOL, asthma control and inhalation technique in adult asthmatic patients. It can be concluded that methylxanthine was most often prescribed drug followed by combination of beta agonists and corticosteroids. Hence, the pharmacist provided intervention could help to enhance the QOL, asthma control and proper inhalation technique among asthmatic patients.

## Figures and Tables

**Table 1:** Demographic patterns of the patients.

Variables	Category	Test group *n* (%)	Control group *n* (%)	Total *n* = 72
Age	18–28	2 (5.6)	3 (8.3)	5 (6.9)
	29–38	7 (19.4)	7 (19.4)	14 (19.4)
	39–48	5 (13.9)	8 (22.2)	13 (18.1)
	49–58	7 (19.4)	4 (11.1)	11 (15.3)
	>59	15 (41.7)	14 (38.8)	29 (40.3)

Gender	Male	19 (59.3)	13 (40.7)	32 (44.4)
	Female	17 (42.5)	23 (57.5)	40 (55.6)

Marital status	Married	25 (69.4)	18 (50)	43 (59.7)
	Unmarried	2 (5.6)	9 (25)	11 (15.3)
	Divorced	2 (5.6)	0 (0)	2 (2.8)
	Widow/widower	7 (19.4)	9 (25)	16 (22.2)

Family type	Nuclear	17 (47.2)	15 (41.6)	32 (44.4)
	Joint	13 (36.1)	14 (38.8)	27 (37.5)
	Extended	6 (16.6)	7 (19.4)	13 (18.0)

Education status	Illiterate	14 (38.9)	15 (41.7)	29 (40.3)
	School level/SEE	11 (30.6)	12 (33.3)	23 (31.9)
	Intermediate	8 (22.2)	6 (16.7)	14 (19.4)
	Bachelor or above	3 (8.3)	3 (8.3)	6 (8.3)

Family source of income	Agriculture	7 (19.4)	5 (13.9)	12 (16.6)
	Family business	4 (11.1)	4 (11.1)	8 (11.1)
	Remmittance	12 (33.3)	9 (25.0)	21 (29.1)
	Private/government job	6 (16.7)	10 (27.8)	16 (22.2)
	Labor/daily wages	2 (5.6)	4 (11.1)	6 (8.3)
	Pension	5 (13.3)	3 (8.3)	8 (11.1)
	Others	0	1 (2.8)	1 (1.3)

Duration of suffering from asthma	Less than 1 month	1 (2.8)	2 (5.6)	3 (4.2)
	6 month	4 (11.1)	4 (11.1)	8 (11.1)
	1 year	5 (13.9)	6 (16.7)	11 (15.3)
	2 years	5 (13.9)	10 (27.8)	15 (20.8)
	More than two years	21 (58.3)	14 (38.9)	35 (48.6)

*n* indicates number of patients and values in the brackets indicate percentage.

**Table 2:** Quality of life of asthmatic patients before and after intervention.

Groups	Mean score of MiniAQLQ		*p* value^a^
	Before intervention	After intervention	
Test group	2.39 ± 0.55	5.64 ± 0.31	0.001^a^
Control group	2.43 ± 0.44	5.41 ± 0.40	0.001^a^
*p* value^b^	0.001^b^	0.001^b^	

^a^ *p* value for differences between before and after intervention in each group.

^b^ *p* value for differences between test and control group. Statistically significant (*p* < 0.05).

**Table 3:** Range of QOL in MiniAQLQ score before and after intervention.

MiniAQLQ		Test group		Control group	
Score	Range	Before intervention (*n*)	After intervention (*n*)	Before intervention (*n*)	After intervention (*n*)
<4	Poor	22	0	21	0
4	Moderate	0	0	0	3
>4	Excellent	3	25	0	18

*n* indicates the number of patients.

**Table 4:** Level of asthma control of asthmatic patients before and after intervention.

Groups	Mean score of ACQ		*p* value^a^
	Before intervention	After intervention	
Test group	3.54 ± 0.42	1.15 ± 0.20	0.001^a^
Control group	3.19 ± 0.66	2.65 ± 1.01	0.099^a^
*p* value^b^	0.001^b^	0.001^b^	

^a^ *p* value for differences between before and after intervention in each groups. ^b^ *p* value for differences between test and control group. Statistically significant (*p* < 0.05).

**Table 5:** Range of asthma control in ACQ score before and after intervention.

ACQ		Test group		Control group	
Score	Range	Before intervention (*n*)	After intervention (*n*)	Before intervention (*n*)	After intervention (*n*)
>1.5	Uncontrolled	25	2	21	17
<1.5	Controlled	0	23	0	4

*n* indicates the number of patients

**Table 6:** Mean score of MDI before and after intervention.

Performance	Mean score ± SD^b^	*p* value
Before intervention	5.68 ± 1.35	0.001^a^
After intervention	7.25 ± 0.57	

^a^ *p* value less than 0.05 is considered statistically significant, ^b^SD Standard Deviation.

**Table 7:** Percentage of patients performing each step correctly.

S.no.	Steps	Before intervention *n* (%)	After intervention *n* (%)
(1)	Remove cap from the mouth-piece of canister, hold upright	15 (93.8)	16 (100)
(2)	For the first use or using after more than 7 days, shake and release one puff into air	11 (68.8)	12 (75.0)
(3)	Stand or sit straight. Breathe out through the mouth	12 (75.0)	14 (87.5)
(4)	Place the mouth-piece between teeth and close lips without leaving any gap	14 (87.5)	16 (100.0)
(5)	Breath in and release one dose with simultaneously breathing in	15 (93.8)	15 (93.8)
(6)	Remove the inhaler and close the mouth immediately	12 (75)	16 (100.0)
(7)	Hold breath for 10 s or if possible	4 (25)	13 (81.3)
(8)	Wait for at least one minute before taking the second dose	8 (50)	4 (87.5)

*n* indicates number of patients and values in the bracket indicate percentage.

**Table 8:** Mean score of DPI before and after intervention.

Performance	Mean score ± SD^b^	*p* value
Before intervention	8.55 ± 0.88	0.001^a^
After intervention	10.44 ± 0.72	

^a^ *p* value less than 0.05 is considered statistically significant. ^b^Standard Deviation.

**Table 9:** Percentage of patients performing each step correctly.

S.no.	Steps	Before intervention *n* (%)	After intervention *n* (%)
(1)	Keep the rotahaler upright	9 (100)	9 (100)
(2)	Insert rotacap with transparent end down	8 (88.9)	9 (100)
(3)	Keep rotahaler horizontal	9 (100)	9 (100)
(4)	Rotate both ends to open capsule	8 (88.9)	8 (88.9)
(5)	Exhale to residual volume	2 (22.2)	8 (88.9)
(6)	Keep rotahaler level and put mouth piece between teeth and lips	9 (100)	9 (100)
(7)	Inhale powder forcefully and deeply	7 (77.8)	8 (88.9)
(8)	Remove rotahaler from the mouth and hold breath for 5 s	4 (44.4)	8 (88.9)
(9)	Exhale away from the mouthpiece	4 (44.4)	9 (100)
(10)	If any powder is left, repeat steps from (1)	9 (100)	9 (100)
(11)	Open the rotahaler and discard the capsule	8 (88.9)	8 (88.9)

*n* indicates number of patients and values in the bracket indicate percentage.

**Table 10:** Measurement of effectiveness of various types of demonstration.

Verbal method	*SA*	*A*	*N*	*D*	*SD*
The leaflet was clear and easy to understand	21 (84)	3 (12)	1 (4)	0	0
I feel comfortable and able to follow	10 (40)	13 (52)	2 (8)	0	0
I feel complete confident in doing my inhaler	15 (60)	9 (36)	1 (4)	0	0
I felt supported and encouraged	21 (84)	4 (16)	0	0	0

*Video aid method*					
The leaflet was clear and easy to understand	25 (100)	0	0	0	0
I feel comfortable and able to follow	21 (84)	4 (16)	0	0	0
I feel complete confident in doing my inhaler	18 (72)	7 (28)	0	0	0
I felt supported and encouraged	21 (84)	3 (12)	0	0	0

Values in brackets indicate percentage. SA: Strongly Agree, A: Agree, N: Neutral, D: Disagree, SD: Strongly Disagree.

**Table 11:** Drugs prescribed in the treatment of asthma.

Drug category	Name of the drugs	Dosage form	Dose	Frequency	No. of Prescription *n* (%)
Methylxanthine	Theophylline, doxofylline, etophylline and theophylline	Tablet	200 mg, 300 mg, 400 mg	OD	44 (24.5%)
Beta 2 agonists + ICS	Salmeterol + fluticasone, salmeterol + beclomethasone	Inhaler	(50 + 250) mcg,(50 + 500) mcg	2 puffs OD	39 (21.7%)
Beta 2 agonists	Salbutamol, salmeterol	Inhaler	200 mcg	2-3 puffs BD	26 (14.5%)
Antibiotics	Macrolides, beta lactam antibiotics	Capsule	500 mg, 500 + 125 mg	TDS for 5 days or 7 days	23 (12.8%)
Anticholinergic	Tiotropium, ipratopium	Inhaler	18 mcg, 20 mcg	2 puffs BD	22 (12.2%)
Inhaled corticosteroids (ICS)	Budesonide	Inhaler	200 mcg	2 puffs OD	17 (9.4%)
Leukotriene antagonists	Montelukast	Tablet	10 mg	OD	8 (4.4%)

Abbreviations: ICS, Inhaled corticosteroids; OD, Once daily; BD, twice daily; mg, milligrams; mcg, micrograms.

## Data Availability

The data is securely stored by the researcher and is available upon request.

## Supplementary Materials

Supplementary Materials Appendix 1: Informed Consent in English Statement by the researcher/person taking consent Appendix 2a Questionnaires for the research Appendix 2b Mini Asthma Quality of Life Questionnaire in English Appendix 3: Checklist for MDIs and DPIs Appendix 4a Feedback Form Appendix 4b DRUG TREATMENT CHART.
