# Supplementary material for: Pharmacist Led Intervention on Inhalation Technique among Asthmatic Patients for Improving Quality of Life in a Private Hospital of Nepal
Source: Pulm Med. 2019 Nov 24;2019:8217901. doi: 10.1155/2019/8217901 (PMC6930739; doi:10.1155/2019/8217901)
Supplement: Supplementary Materials — Appendix 1: Informed Consent in English Statement by the researcher/person taking consent Appendix 2a Questionnaires for the research Appendix 2b Mini Asthma Quality of Life Questionnaire in English Appendix 3: Checklist for MDIs and DPIs Appendix 4a Feedback Form Appendix 4b DRUG TREATMENT CHART. [file 8217901.f1.pdf]

Appendix 1: Informed Consent in English

I have read the foregoing information, or it has been read to me. I have had the opportunity to ask questions about it and any questions I have been asked have been answered to my satisfaction. I consent voluntarily to be a participant in this study.

Name of Participant__________________

Signature of Participant ___________________

Date ___________________________

Day/month/year

If illiterate [1]

I have witnessed the accurate reading of the consent form to the potential participant, and the individual has had the opportunity to ask questions. I confirm that the individual has given consent freely.

Name of witness____________

Signature of witness _____________

Date ________________________

Day/month/year

Thumb print of participant

[1] A literate witness must sign (if possible, this person should be selected by the participant and should have no connection to the research team). Participants who are illiterate should include their thumb print as well.

Statement by the researcher/person taking consent

I have accurately read out the information sheet to the potential participant, and to the best of my ability made sure that the participant understands that the following will be done:

1. I understand that even if I agree to participate now, I can withdraw at any time or refuse to answer any question without any consequences of any kind.
2. I understand that all information I provide for this study will be treated confidentially.
3. I understand that in any report on the results of this research my identity will remain anonymous.

I confirm that the participant was given an opportunity to ask questions about the study, and all the questions asked by the participant have been answered correctly and to the best of my ability. I confirm that the individual has not been coerced into giving consent, and the consent has been given freely and voluntarily.

A copy of this ICF has been provided to the participant.

Name of Researcher/person taking the consent________________________

Signature of Researcher /person taking the consent__________________________

Date ___________________________

Day/month/year

Appendix 1 Informed Consent

सहमतिपत्र

मितिः

<u>विषयः अनुसन्धान परियोजनामा सहभागिताको लागि अनुरोध</u>

आदरणीय सहभागीज्यू,

नमस्कार,

पोखरा विश्वविद्यालय, पोखराको फार्मेसी विभाग र क्रिमसन अस्पताल, रुपन्देहीको स्वीकृतिमा फार्मासिस्टको कारणले गर्दा दमको विरामीमा जीवनगुणस्तर सुधार्न, दम नियन्त्रण गर्न र इन्हेलर प्रविधिको उचित प्रयोग सम्बन्धमा गर्न लागिएको यस अनुसन्धान कार्यमा म यहाँको स्वेच्छिक सहभागिताको लागि विनम्र अनुरोध गर्दछु।

यस सहमतिपत्रलाई सावधानीपूर्वक पढ्नुहोस्। तपाई यस अनुसन्धानमा सहभागि हुन स्वतन्त्र हुनुहुन्छ।यदि तपाईलाई यस अनुसन्धान वा यस सहमतिपत्र बारेमा कुनै प्रस्नहरु छन् भने कृपया निर्धक्तापूर्वक सोध्नुहोला। तपाईंलाई यस अनुसन्धानमा सहभागी हुन चाहनुहुन्छ भने तपाईंलाई यो सहमतिपत्रमा हस्ताक्षर गर्नुपर्नेछ। जसको एक प्रतिलिपी तपाईंलाई पनि प्रदान गराइनेछ।

पृष्ठभूमि

दम एउटा यस्तो रोग हो जसमा फोक्सो को वायुनली संकीर्ण भई सास फेर्न गार्हो हुन्छ। यो रोग हाम्रो नेपाली समाजमा धेरै वर्ष देखी छ। पहिलेपहिले दमका रोगीहरुले उपचारको कमिले गर्दा अकालमै ज्यान गुमाउनुपर्थ्यो। तर अहिले परिस्थिती फरक छ। विज्ञान र प्रविधीले गरेको विकास सँगै दमको सफल उपचार सम्भव छ। यस उपचार प्रकृयामा फार्मासिस्टहरुले महत्वपूर्ण भूमिका प्रदान गरेका छन। नेपालमा सन् २०१७ मा गरेको एक अध्ययन अनुसार कुल मृत्युदरमा २.०५% दमको करणले मृत्यु हुने गरेको छ। दमको करणले मृत्यु हुनेमा नेपाल २६औँ स्थानमा पर्दछ।

अनुसन्धानको उद्देश्यहरु

- दमको विरामीको जीवनगुणस्तर नापन गर्न
- दमको विरामीको दम नियन्त्रण नापन गर्न
- दम को विरामीलाई इन्हेलर प्रविधीको सहि प्रयोग गर्न सिकाउन
- दमको विरामीमा औषधी व्यवस्थापन हेर्न

अनुसन्धानको सम्भावित फाइदा र बेफाइदा

- फाइदा

वर्तमान अध्ययनले विरामीहरुलाई दमबारे जानकारी प्रदान गर्नुको साथै उनिहरुलाई दमको जटिलता बाट जोगाउन र मृत्युबाट बचाउन सकिनेछ।

- बेफाइदा

तपाईंलाई केही प्रश्नहरुको जवाफ दिन सहज महसुस नहुनसक्छ, कृपया यो प्रश्नहरुको जवाफ दिई हामीलाई सहयोग गरिदिनुहोला।

गोपनियता

तपाईबाट प्रप्त जानकारी गोप्य राखिने छ तथा यस अध्ययनका लागि मात्र प्रयोग गरिनेछ। यस अनुसन्धानमा तपाईको नामलाई कुनै चिन्ह अथवा अंकको रूपमा लेखिनेछ। तपाईलाई माथि भनिएकोजस्तै तपाईको जवाफहरुमा तपाईको व्यक्तिगत सुचनाहरुको समावेस गरिने छैन यो सब परिचय नम्बरको आधारमा गरिनेछ र डेटा केबल शोधकर्ताहरुद्वारा मास्टर थेसिसको लागि उपयोग गरिनेछ। तपाईं कुनै पनि कारण बताई कुनै पनि समयमा आफ्नो सहभागीता लिनबाट फिर्ता लिन सक्नुहुन्छ। तपाईंको बारेमा एकत्रित गरिएको डाटा मेटिनेछ, बाहेक यो पहिले नै समावेश भए तापनि, यदि तपाईं अध्ययनबाट नै हट्न चाहनुहुन्छ भने तपाईंले चिकित्सकबाट लिइने सल्लाह तथा उपचारमा कुनै असर पर्ने छैन अन्तिममा तपाईंलाई इन्हेलर प्रविधीको सही प्रयोग बारे परामर्श दिइनेछ।

यदि तपाई संग कुनै प्रश्नहरु छन् भने तपाईले अहिले अथवा अध्ययनको क्रममा सोध्न सक्नुहुनेछ अन्य जानकारीका लागि कृपया उल्लेखित ठेगानामा संपर्क गर्नुहोला।

अनुसन्धानकर्ताको नाम र संपर्क ठेगाना:

अनिता यादव

M.Pharm (Clinical Pharmacy)अध्ययनरत

पोखरा विश्वविद्यालय, पोखरा

९८०७४७५५६२

anita.yadav2047@gmail.com

सहमति सटिर्फिकेट

अनुसन्धानको विषय

“PHARMACIST LED INTERVENTION ON INHALATION TECHNIQUE AMONG ASTHMATIC PATIENTS FOR IMPROVING QUALITY OF LIFE IN A PRIVATE HOSPITAL OF NEPAL”

अनुसन्धान अन्वेषकको नाम

अनिता यादव

पोखरा विश्वविद्यालय

मैले अग्रिम जानकारी पढेको छु वा मलाई पढेर सुनाईएको छ। मैले यसमा प्रश्न सोध्नने मौका पाएको छु। सोधिएका प्रश्नहरुको चित्त बुझ्दा उत्तरहरु पाईएकोछ। म यसमा स्वेच्छाले भाग लिन गईरहेकोछु।

म बाट संकलन गरिएको जानकारी गोप्य राखी अनुसन्धान प्रयोजनको लागि मात्र प्रयोग गरिने साथै यस अनुसन्धानमा सहभागि भए बापत कुनै जोखिम र वितिय लाभ नहुने जानकारी पनि गराइको छ। मलाई उप्लब्ध सम्पूर्ण जानकारीबाट सन्तुष्ट भई यस अनुसन्धानमा सहभागि भएको र मैले प्रदान गरेको जानकारी उक्त अनुसन्धानमा प्रयोग गर्नको लागि स्वीकृति दिएको व्यहोरा उल्लेख गर्दछु।

सहभागिको नामः____

सहभागिको हस्ताक्षरः____

मिति:____________________________

(दिन/ महिना/ वर्ष)

अशिक्षितको लागि

सहभागिको लागि सहमतिपत्र ठीक संग पढिएको / बुझाइएको म साक्षी छु। सहभागिलाई जिज्ञासा रख्ने मौका दिइएको छ र म यकिन गर्छु कि सह्भागिले सो इच्छाले दिएका हुन्।

साक्षीको नामः____

सहभागिको औँठा छाप

साक्षीको हस्ताक्षरः________________________________

मितिः__________________________

(दिन/ महिना/ वर्ष)

दायाँ	बायाँ

<u>सहमति लिने अनुसन्धानकर्ताको बयान</u>

मैले सहमति पत्र ठीकसंग सहभागिको लागि पढेकोछु र मेरो क्षमता अनुसार नीजलाई बुझाउन प्रयास गरेको छु । म यकिन गर्छु कि सहभागिलाई जिज्ञासा रख्न मौका दिइएको छ र सबै जिज्ञासाहरुको उत्तर मेरो दक्षताअनुसार सहि रुपमा दिएको छु। म यकिन गर्छु कि सहभागिलाई सहमति दिन कुनै करकाप गरिएको छैन र स्वतन्त्रर स्वइच्छाले दिइएको हो।

सहमति लिने अनुसन्धानकर्ताको

नामः____________________________________

हस्ताक्षरः____

मितिः____(दिन/ महिना/ वर्ष)

Appendix 2a Questionnaires for the research

Questionnaires for the research(अनुसन्धानमा सोधिने प्रश्नहरु)

Participant Code:<br>सहभागि नं:	Date:<br>मितिः	Age:<br>उमेर:
Name of Participant:<br>सहभागिको नाम:		
Address of participant:<br>सहभागिको ठेगाना:		
Contact No:<br>सम्पर्क न:		

Please write the (√) on the suitable answer.(ठीक उत्तरमा (√) चिन्ह लगाउनुहोस्।)

May I begin your interview?(के म तपाईको अन्तरवार्ता सुरु गर्न सक्छु?)

Yes. ☐ No. ☐

S.N. क्रम न:	Questions)प्रश्न)	Response(प्रतिक्रिया)
1.	तपाईको उमेर कति भयो?<br>How old are you?	1. 18-28(१८-२८)<br>2. 29-38(२९-३८)<br>3. 39-48(३९-४८)<br>4. 49-58(४९-५८)<br>5. >59(>५९)
2.	तपाईको लिङ्गके हो?<br>What is your gender?	1. Male(पुरुष)<br>2. Female(महिला)<br>3. Other(अन्य)
3.	तपाईको वैवाहिक अवस्थाके छ?<br>What is your marital status?	1. Married(विवाहित)<br>2. Unmarried(अविवाहित)<br>3. Divorced)छोडपत्र(<br>4. Widow/Widower(विधवा/विधुर)

4.	तपाईं क्स्तो परिवारमा बस्नु हुन्छ ?In Which type of family you stay?	1. Nuclear(एक्ल)<br>2. Joint)संयुक्त(<br>3. Extended)बृहत)
5.	तपाईको शैक्षिकयोग्यताकति हो?<br>What is your educational qualification?	1. Illiterate(अनपढ)<br>2. School Level/SLC(स्कुल तह)<br>3. Intermediate(कलेज तह)<br>4. Bachelor or above(स्नातक तह औँ माथिल्लो)
6.	तपाईको परिवारको आयश्रोतको माध्यम के हो?<br>What is your family source of income?	1. Agriculture/Farming(कृषि)<br>2. Family Business(घरेलु व्यवसाय)<br>3. Remittance)वैदेशिक(<br>4. Private/Government Job(गैर-सरकारी /सरकारी(<br>5. Labor/Daily wages(श्रमिक)<br>6. Pension(पेन्सन)<br>7. Others (अन्य)
7.	तपाईंलाई दम कहिले देखि छ ?<br>(From when you have been suffering from asthma?)	1. Less than 1 month(१ महिना भन्दा कम)<br>2. 6 month (६ महिना)<br>3. 1 years (१ वर्ष)<br>4. 2 years (२वर्ष)<br>5. More than 2 years (२ वर्ष भन्दा बढी)

Thank you for your participation.(तपाईको सहभागिताको लागि धन्यवाद)

Appendix 2b Mini Asthma Quality of Life Questionnaire in English

Please complete all questions by circling the number that best describes how you have been during the last 2 weeks as a result of your asthma.

In general, how much of the time during the last 2 weeks did you:

S.no	Questions	All of the time	Most of the Time	A Good Bit of the Time	Some of the Time	A Little of the Time	Hardly Any of the Time	None of the Time
1	Feel SHORT OF BREATH as a result of your asthma?	1	2	3	4	5	6	7
2.	Feel bothered by or have to avoid DUST in the environment?	1	2	3	4	5	6	7
3.	Feel FRUSTRATED as a result of your asthma?	1	2	3	4	5	6	7
4.	Feel bothered by COUGHING?	1	2	3	4	5	6	7
5.	Feel AFRAID OF NOT HAVING YOUR ASTHMA MEDICATION AVAILABLE?	1	2	3	4	5	6	7
6.	Experience a feeling of CHEST TIGHTNESS or CHEST HEAVINESS?	1	2	3	4	5	6	7
7.	Feel bothered by or have to avoid CIGARETTE SMOKE in the environment?	1	2	3	4	5	6	7
8.	Have DIFFICULTY GETTING A GOOD NIGHT'S SLEEP as a result of your asthma?	1	2	3	4	5	6	7

9.	Feel CONCERNED ABOUT HAVING ASTHMA?	1	2	3	4	5	6	7
10.	Experience a WHEEZE in your chest?	1	2	3	4	5	6	7
11.	Feel bothered by or have to avoid going outside because of WEATHER OR AIR POLLUTION?	1	2	3	4	5	6	7
12.	STRENUOUS ACTIVITIES (such as hurrying, exercising, running up stairs, sports)	1	2	3	4	5	6	7
13.	MODERATE ACTIVITIES (such as walking, housework, gardening, shopping, climbing stairs)	1	2	3	4	5	6	7
14.	SOCIAL ACTIVITIES (such as talking, playing with pets/children, visiting friends/relatives)	1	2	3	4	5	6	7
15.	WORK-RELATED ACTIVITIES* (tasks you have to do at work)	1	2	3	4	5	6	7

Mini Asthma Quality of Life Questionnaire in Hindi Version

दमा जीवन की गुणवत्ता प्रश्नावली**(संक्षिप्त)**
(HINDI VERSION)
स्वयं भरी गई

रोगी परिचय ______________________________

तिथि ___________________________________

पिछले दो सप्ताहों के दौरान आप अपने दमारोग के कारण इन क्रियाओं (activities) को करने में कितने आप कितने सीमित रहे हैं, इसका सबसे अच्छा वर्णन करने वाली संख्या को गोल घेर कर कृपया **सभी** प्रश्नों का उत्तर दीजिए।

आमतौर पर, कुल मिलाकर पिछले दो सप्ताहों के दौरान **कितने समय** आपको

	सारे समय	अधिकतर समय	काफी समय	थोड़े समय	बहुत थोडे समय	मुश्किल से कभी	कभी नहीं
1. अपने दमारोग के फलस्वरूप **साँस फूलने का लक्षण** महसूस हुआ है ?	1	2	3	4	5	6	7
2. **वातावरण में धूल की वजह से** किसी जगह जाने में परेशानी महसूस हुई या उस जगह से दूर रहना पड़ा ?	1	2	3	4	5	6	7
3. अपने दमा रोग के कारण **निराशा** महसूस हुई है ?	1	2	3	4	5	6	7
4. **खाँसी** की वजह से परेशानी महसूस हुई ?	1	2	3	4	5	6	7
5. **यह भय** महसूस हुआ कि **दमा की अपनी दवाई आपको न मिल पाऐगी ?**	1	2	3	4	5	6	7
6. दमे की वजह से **छाती में जकड़न या भारीपन** महसूस हुआ ?	1	2	3	4	5	6	7

दमा जीवन की गुणवत्ता प्रश्नावली**(संक्षिप्त)**
(HINDI VERSION)
स्वयं भरी गई

रोगी परिचय ______________________

तिथि ______________________

आमतौर पर, कुल मिलाकर पिछले दो सप्ताहों के दौरान **कितने समय** आपको

	सारे समय	अधिकतर समय	काफी समय	थोड़े समय	बहुत थोडे समय	मुश्किल से कभी	कभी नहीं
7. **वातावरण में सिगरेट के धुंए की वजह से** किसी जगह जाने में परेशानी महसूस हुई या उस जगह से दूर रहना पड़ा ?	1	2	3	4	5	6	7
8. दमे की वजह से **रात को अच्छी नींद नहीं आई** ?	1	2	3	4	5	6	7
9. **दमारोग ग्रस्त होने के बारे में चिन्ता** महसूस हुई है ?	1	2	3	4	5	6	7
10. अपनी छाती में **सीटी की सी आवाज** महसूस हुई है?	1	2	3	4	5	6	7
11. **वायु प्रदूषण और मौसम की वजह से** बाहर जाने में परेशानी महसूस हुई या उस से बचना पड़ा	1	2	3	4	5	6	7

पिछले दो सप्ताहों के दौरान अपने दमारोग के कारण आप इन क्रियाओं को करने में कितने सीमित हुए हैं ?

	पूर्णतः सीमित	बहुत अधिक सीमित	अधिक सीमित	काफी सीमित	कुछ सीमित	बहुत कम सीमित	बिल्कुल सीमित नही
12. **भारी क्रियाएँ** (जैसे कि जल्दबाजी, कसरत, तेजी से जीने पर चढ़ना, खेलकूद) करने में	1	2	3	4	5	6	7

दमा जीवन की गुणवत्ता प्रश्नावली**(संक्षिप्त)**
(HINDI VERSION)
स्वयं भरी गई

रोगी परिचय ____________________

तिथि ____________________

पिछले दो सप्ताहों के दौरान अपने दमारोग के कारण आप इन क्रियाओं को करने में कितने सीमित हुए हैं ?

	पूर्णतः सीमित	बहुत अधिक सीमित	अधिक सीमित	काफी सीमित	कुछ सीमित	बहुत कम सीमित	बिल्कुल सीमित नही
13. **मध्यम दर्जे की क्रियाएँ** (जैसे कि चलना , धरेलू कामकाज , बागवानी, खरीदारी, सीढ़ियाँ चढ़ना) करने में	1	2	3	4	5	6	7
14. **सामाजिक क्रियाएँ** (जैसे कि बातचीत करना, पालतू पशुओं / बच्चों के साथ खेलना, मित्रों, रिश्तेदारों से मिलने जाना)	1	2	3	4	5	6	7
15. **व्यवसाय सम्बन्धी क्रियाएँ** (काम काज सम्बन्धी **क्रियाएँ***)	1	2	3	4	5	6	7

***** *यदि आप नौकरी नहीं करते या स्वयं का कोई व्यवसाय नहीं करते हैं तो ये वे कार्य होने चाहिए जो आप अधिकांष दिनों में करते हैं ।*

अधिकार क्षेत्र संकेत पद्वति

लक्षण : 1, 4, 6, 8, 10

क्रियाकलाप सीमित होना : 12, 13, 14, 15

भावनात्मक कार्य : 3, 5, 9

वातावरण संबंधी तत्त्व : 2, 7, 11

Asthma Control Test Questionnaire in English

Patient ID……………….

Date……………………………….

Please answer questions 1 - 6.

Circle the numberof the response that best describes how you have been during the past week.

1. On average, during the past week, how often were you **woken by your asthma** during the night?

0 Never

1 Hardlyever

2 A fewtimes

3 Several times

4 Manytimes

5 A greatmany times

6 Unable to sleep because of asthma

2. On average,duringthepast week, how **bad were your asthma symptoms when you woke up** in the morning?

0 No symptoms

1 Very mild symptoms

2 Mild symptoms

3 Moderates ymptoms

4 Quite severe symptoms

5 Severe symptoms

6 Very severe symptoms

3. In general,during the pastweek,how **limited were you in youra ctivities** because of your asthma?

0 Not limitedat all

1 Veryslightlylimited

2 Slightlylimited

3 Moderately limited
4 Verylimited
5 Extremelylimited
Totally limited

4. In general, during the past week, how much **shortness of breath** did you experience because of your asthma?

0 None
1 A very little
2 Alittle
3 A moderate amount
4 Quitea lot
5 A great deal
6 A verygreatdeal

5. In general, during the past week, how much of the time did you **wheeze?**

0 Not at all
1 Hardly anyof thetime
2 A little ofthetime
3 A moderate amount of thetime
4 Alot of thetime
5 Mostofthetime
6 All the time

6. On average, during the past week, how many **puffs/inhalations of short-acting bronchodilator** (eg.Ventolin/Bricanyl) have you used each day? *(If you are not sure how to answer this question, please ask for help)*

0 None
1 1-2puffs/ inhalations most days
2 3-4puffs/inhalations most days

3 5-8puffs/inhalations most days

4 9- 12 puffs/inhalations most days

5 13 - 16 puffs/inhalations most days

6 Morethan16 puffs/inhalations most days

To be completed by a member of the clinic staff

7. FEV_1pre-bronchodilator: ....

FEV_1predicted: ....

FEV_1%predicted: ....

(Record actual values on the dotted lines and score the FEV_1 %predicted in the next column)

0 >95%predicted

1 95-90%

2 89-80%

3 79-70%

4 69-60%

5 59-50%

6 <50%predicted

Asthma Control Test Questionnaire in Hindi Version

दमा नियंत्रण प्रश्नावली ©
(HINDI VERSION FOR INDIA)

रोगी परिचय ____________________

अतिथि / दिनांक ____________________

कृपया 1 – 6 प्रश्नों के उत्तर दें।

जवाब की उस संख्या को **घेरें** जो सबसे बेहतर रूप से ये बयान करती हो कि आप पिछले हफ्ते में कैसे रहें।

1. पिछले हफ्ते में, औसतन कितनी बार **दमे की वजह से आप रात में जग** गए?
   - 0 कभी नहीं
   - 1 शायद ही कभी
   - 2 कुछ बार
   - 3 कई बार
   - 4 बहुत बार
   - 5 अत्यधिक बार
   - 6 दमे की वजह से सो नहीं पाता

2. पिछले हफ्ते में, औसतन **जब आप सवेरे उठते थे तो दमे के लक्षण कितने ज़्यादा खराब थे**?
   - 0 कोई लक्षण नहीं
   - 1 बहुत हल्के लक्षण
   - 2 हल्के लक्षण
   - 3 कुछ (मध्यम) लक्षण
   - 4 थोड़े गंभीर लक्षण
   - 5 गंभीर लक्षण
   - 6 बहुत गंभीर लक्षण

3. पिछले हफ्ते में, सामान्यत: दमे की वजह से आप **अपनी क्रियाओं** (जैसे घरेलू, स्कूल संबंधी, कार्य संबंधी इत्यादि) **में कितने सीमित रहे हैं**?
   - 0 बिलकुल सीमित नहीं
   - 1 बहुत थोड़े सीमित
   - 2 थोड़े सीमित
   - 3 कुछ (मध्यम) सीमित
   - 4 बहुत ज़्यादा सीमित
   - 5 अत्यधिक सीमित
   - 6 संपूर्णतया सीमित

4. पिछले हफ्ते में, सामान्यत: दमे की वजह से आपको **साँस फूलने** का अनुभव कितना हुआ?
   - 0 बिल्कुल नहीं
   - 1 बहुत कम
   - 2 कम
   - 3 कुछ (मध्यम) ज़्यादा
   - 4 बहुत ज़्यादा
   - 5 बहुत बहुत ज़्यादा
   - 6 अत्यधिक ज़्यादा

रोगी परिचय ____________________

अतिथि / दिनांक____________________

5. पिछले हफ्ते में सामान्यत: कितने समय **साँस में घरघराहट हुई?**

0 कभी नहीं
1 शायद ही कुछ समय
2 कुछ समय तक
3 कुछ (मध्यम) समय
4 बहुत समय
5 लगभग हर समय
6 हर समय

6. पिछले हफ्ते में, औसतन एक दिन में कितनी बार आपको **कम देर तक असर करने वाली साँस की दवा (जैसे Ventolin/Bricanyl) का कश (puffs/inhalations)** लेना पड़ा?
(अगर आप उत्तर देने के बारे में निश्चित नहीं है तो कृपया मदद माँगें)

0 बिल्कुल नहीं
1 अधिकतर दिन 1 - 2 पफ / इनहेलेशन
2 अधिकतर दिन 3 - 4 पफ / इनहेलेशन
3 अधिकतर दिन 5 - 8 पफ / इनहेलेशन
4 अधिकतर दिन 9 - 12 पफ / इनहेलेशन
5 अधिकतर दिन13 - 16 पफ / इनहेलेशन
6 अधिकतर दिन 16 से ज़्यादा पफ / इनहेलेशन

स्वास्थ्य कर्मचारी द्वारा पूरा किया जाएगा

7. FEV_1 प्री-ब्रोन्कोडायलेटर (pre-bronchodilator)................................

FEV_1 संभावित...................................

FEV_1% संभावित................................
(वास्तविक मान को डॉटेड (dotted) रेखा पर लिखें और FEV1 के संभावित प्रतिशत को अगले कॉलम (column) में लिखें)

0 > 95% संभावित
1 95 - 90%
2 89 - 80%
3 79 - 70%
4 69 - 60%
5 59 - 50%
6 < 50% संभावित

Appendix 3: Checklist for MDIs and DPIs

Patient ID………………. Date..........................

MDIs (Metered Dose Inhalers)

S.No.	Steps	Before Intervention	After Intervention
1	Remove cap from the mouth-piece of canister, hold upright		
2	For the first use or using after more than 7 days, shake and release one puff into air		
3	Stand or sit straight. Breathe out through the mouth		
4	Place the mouth-piece between teeth and close lips without leaving any gap		
5	Breath in and release one dose with simultaneously breathing in		
6	Remove the inhaler and close the mouth immediately		
7	Hold breath for 10 seconds or if possible		
8	Wait for at least one minute before taking the second dose.		
Total Score			

For DPIs (Dry Powder Inhalers)

S.No.	Steps	Before Intervention	After Intervention
1	Keep the rotahaler upright		
2	Insert rotacap with transparent end down		
3	Keep rotahaler horizontal		
4	Rotate both ends to open capsule		
5	Exhale to residual volume		
6	Keep rotahaler level and put mouth piece between teeth and lips		
7	Inhale powder forcefully and deeply		
8	Remove rotahaler from the mouth and hold breath for 5 seconds		
9	Exhale away from the mouthpiece		
10	If any powder is left, repeat steps from 1		
11	Open the rotahaler and discard the capsule.		
Total Score			

Appendix 4a Feedback Form (प्रतिक्रिया फारम)

Verbal method(मौखिक विधि)	Video Method(भिडियो विधि)

Patient Name (विरामीको नाम)	Inhaler Type (इन्हेलर प्रकार)	Date (मिति)

Your Objective feedback will be greatly appreciated. (तपाईंको प्रतिकृयाको अत्याधिक प्रशंसा गरिनेछ।)

Please answer all the statement according to the following 1 to 5 scale. (कृपया 1 देखि 5 मापन अनुसार सबै बयानको जवाफ दिनुहोस्।)

1=Strongly Disagree (SD) (दृढ असहमत)

2=Disagree (D) (असहमत)

3=Neutral (N) (तटस्थ)

4=Agree (A) (सहमत)

5=Strongly Agree (SA) (दृढतापूर्वक सहमत)

**Verbal Method** (मौखिक विधि)	SA	A	N	D	SD
The leaflet was clear and easy to understand.(पत्रक स्पष्ट र बुझ्न सजिलो थियो।)	5<br>५	4<br>४	3<br>३	2<br>२	1<br>१
I feel comfortable and able to follow. (मलाई सहज महसुस भयो र म यसलाई पछ्याउन सक्छु।)	5<br>५	4<br>४	3<br>३	2<br>२	1<br>१
I feel complete confident in doing my inhaler.(म इन्हेलर पूर्णतया राम्रोसँग प्रयोग गर्छु भनेर महसुस गर्छु।)	5<br>५	4<br>४	3<br>३	2<br>२	1<br>१
I felt supported and encouraged.(म समर्थित र प्रोत्साहित महसुस गर्दै छु।)	5<br>५	4<br>४	3<br>३	2<br>२	1<br>१

**Video Aid Method** (भिडियो विधि)	SA	A	N	D	SD
The content of video was clear and easy to understand.(पत्रक स्पष्ट र बुझ्न सजिलो थियो।)	5<br>५	4<br>४	3<br>३	2<br>२	1<br>१

I feel comfortable and able to follow. (मलाई सहज महसुस भयो र म यसलाई पछ्याउन सक्छु।)	5<br>५	4<br>४	3<br>३	2<br>२	1<br>१
I feel complete confident in doing my inhaler.(म इन्हेलर पूर्णतया राम्रोसँग प्रयोग गर्छु भनेर महसुस गर्छु।)	5<br>५	4<br>४	3<br>३	2<br>२	1<br>१
I felt supported and encouraged.(म समर्थित र प्रोत्साहित महसुस गर्दै छु।)	5<br>५	4<br>४	3<br>३	2<br>२	1<br>१

□□Which method was easy to understand? (तपाईंलाई बुझ्न कुन तरिका सजिलो थियो?)

____

□□Out of two which method you prefer to have? (यि दुई मध्ये कुन विधी तपाईं रुचाउनुहुन्छ?)

____

Please return your completed feedback form to counselor.

(कृपया तपाईंको पूर्ण प्रतिक्रिया फारम सल्लाहकारलाई फर्काउनुहोस्।)

Appendix 4b DRUG TREATMENT CHART

Patient ID: **Date:**

S.No	Brand Name	Generic Name	Drug Category	Dose	Route	Frequency	Remarks

Leaflet

दम भनेको के हो?

दम एउटा यस्तो रोग हो जसले तपाईंको फोक्सोको वायुमार्ग संकीर्ण बनाउँछ र खकार उत्पादन गर्छ। त्यसैले दम भएको अवस्थामा साँस फेर्न गाह्रो हुन्छ। दम आक्रमण गर्न सक्ने चिजहरुमा एलर्जी, चिसो, भाईरस, औषधी, धुलोहरु, रसायन, व्यायाम वा भावनाहरु समावेश छन्।

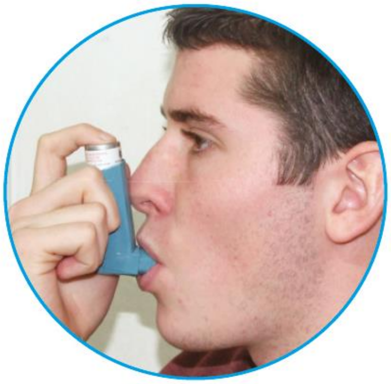

दमबारे जानकारी

लक्षणहरू

- सामान्य भन्दा छिटो साँस चल्नु र साँस फेर्न कठिनाई हुनु।
- स्वा स्वा हुनु, अथवा साँसफेर्दा आवाज आउँनु।
- खोकी, जुन चाँही राती वा विहान् गम्भिर हुनु।
- छातीमा जकडन वा कसाव
- धड्कन तेज हुनु
- घाँटी चिलाउनु वा दुख्नु।
- थकान हुनु।
- यदि तपाईंको चिकित्सकले पीक फ्लो मिटर उपयोग गर्न निर्देष दिनुभएको छ, भने त्यसको मानमा गिरावट आउँनु।

तपाईंलाई दमको अरु लक्षणहरु हुन सक्छ यदि

- तपाईंलाई एलर्जी छ।
- तपाईंको परिवारको कोही सदस्यलाई दम छ।
- वायुप्रदुषण प्रति तपाईं संवेदनशील हुनुहुन्छ।
- धूवाँले प्रभावित गर्छ।
- तनवग्रस्त हुनुहुन्छ।

दम आक्रमणबाट बच्चने उपायहरु

- दमको औषधी सधैं आफुसँग राख्नुहोस्।
- चुरोट र सिगारको धुँवाबाट बच्चनुहोस्।
- जुन खानेकुराले तपाईंलाई दमको लक्षण बढाउँछ, त्यसको सेवन नगर्नुहोस्।
- चिसोबाट बच्चुहोस् वा रुघा-खोकी लागेका व्याक्तिहरुबाट टाढा रहनुहोस्।
- आफ्नो फोक्सो स्वास्थ्य राख्न अनि व्यायम गर्न चिकित्सकसँग सल्लाह लिनुहोस्।
- तनाव कम लिनुहोस्।

आफ्नो चिकित्सकसँग संपर्क गर्नुहोस्

- साँस लिन गाह्रो भइरहेको छ।
- छातीमा दुखाई छ।
- होठ र नङ निलो भएको अवस्थामा।
- ज्वरो आएको बेलामा।
- तपाईंलाई खोकी लागिरहेको र साँसमा घरघराहट भएको अवस्थामा।
- यदि तपाईंलाई चिकित्सकले निर्देष गरेको भन्दा बढी औषधी आवश्यकता छ जस्तो लागेमा।
- तपाईंको खकार सेतो अथवा पारदर्शी नभएर गाढा आइरहेको छ वा खकार निकाल्न गाह्रो भएको अवस्थामा।
- यदि तपाईंले सामान्य गतिविधी गर्न गाह्रो भएको अवस्थामा।

आफ्नो देखभालमा यी कुराहरु समावेश गर्नुहोस्

- हर एक वा दुई घण्टामा एउटा ठुलो गिलास तरल पदार्थ पिउँनुहोस्। यसले तपाईंको खकार पातलो रख्न मद्दत पूर्याउँछ किनभने पातलो खकार खोकी आउँदा सजिलै निस्किन्छ र तपाईंको फोक्सोमा सुजन कम गर्छ।
- पानी, फलफुलको रस, चिया, सूप जस्तो तरल

पदार्थ सेवन गर्नु राम्रो हुन्छ।

- जब साँसमा घरघराहट हुन्छ तब दूध वा दूधजन्य पदार्थ सेवन नगर्नुहोस् किनभने यसले तपाईंको खकार गाढा बनाउँछ।

इन्हेलर कसरी सही तरिकाले प्रयोग गर्ने?

(१) प्रयोग गर्नुभन्दा पहिले इन्हेलरलाई राम्ररी हल्लाउँनुहोस्। (३-४ पटक)

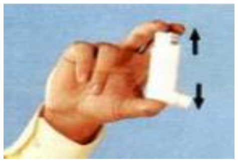

(२) आफ्नो इन्हेलरको बिर्को निकाल्नुहोस्।

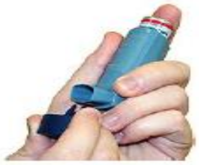

(३) बिस्तारै साँस पूर्णतया बाहिर फाल्नुहोस्।

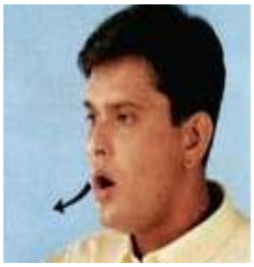

(४) सिधा उभिनुहोस् र आफ्नो टाउको पछाडितिर झुकाउँनुहोस्।

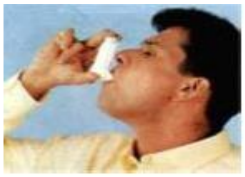

(५) आफ्नो दाँतको बिचमा माउथपिसलाई राख्नुहोस् र ओँठ बन्द गर्नुहोस्।

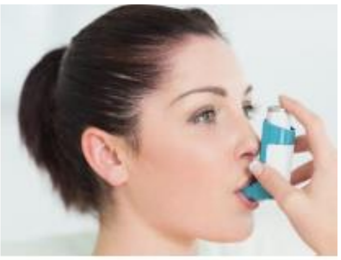

(६) इन्हेलरको माथिल्लो भाग दबाउनुहोस् र साँसभित्र तान्नुहोस्।

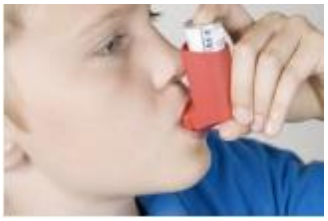

(७) श्वासलाई १० सेकेन्ड सम्म रोक्नुहोस् र साँस बाहिर फाल्नुहोस्।

याद गर्नुपर्ने कुराहरु

- सिधा उभिएर वा उभिन नसके सिधा बसेर इन्हेलर प्रयोग गर्नुपर्छ।
- इन्हेलर लिदा टाउको हल्का पछाडितिर ढलकाउँनुपर्छ।
- अरुलाई आफ्नो इन्हेलर प्रयोग गर्न दिनुहुँदैन।
- प्रयोग पछि मुख पानीले कुल्ला गर्नुपर्छ।
- तपाईंको लक्षणहरु सुधार भएन भने चिकित्सकलाई भेट्नुहोस्।

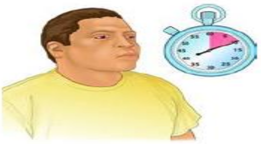

दममा जीवन गुणस्तर र नियन्त्लणबढाउन यी नियमहरु पालन गर्नुहोस्

(१) खानपीन:

दममा के खान हुन्छ?

- दममा ओमेगा थ्री फैटी एसीडयुक्त माछा खानुपर्छ।
- दममा हरियो सागसब्जी खानुपर्छ।
- दममा दूध पिउँनुपर्छ।
- दममा गाजर खानुपर्छ।

- दममा फलफूल खानुपर्छ।
- दममा भिटामिन डी युक्त खानेकुरा खानुपर्छ।

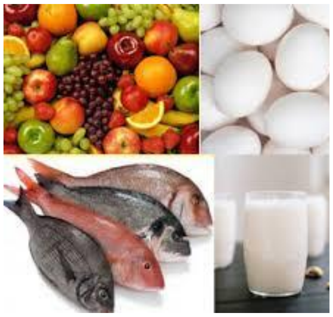

दममा खान नहुने कुराहरु

- दममा तेलमा फ्राई गरेका चिल्लो खानेकुराहरु खानुहुँदैन।
- दममा पनीर र मशरूम खानुहुँदैन।
- अधिक नुन नखानुहोस्।
- प्रशोधन गरिएको खाना नखानुहोस्।
- शराब वा रक्सी को सेवन नगर्नुहोस्।

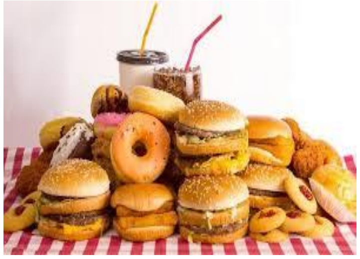

(२) इन्फ्लूएंजा र निमोनियाका लागि टीकाकरण प्राप्त गर्नुपर्छ।

(३) व्यायाम

- व्यायाम गर्दा इनहेलर संगै राख्नुपर्छ।
- व्यायाम विशेषः श्वासप्रश्वास सम्बन्धित हुनुपर्छ।
- जटिल व्यायाम नगर्नुहोस्।
- अत्यन्त उच्च वा अत्यन्त कम तापमानमा व्यायाम नगर्नुहोस्।

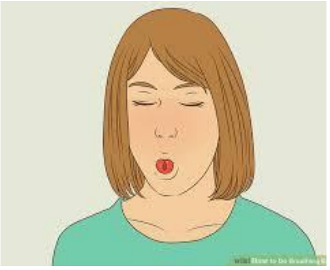

(४) धुम्रपान

- दम का विरामीले धुम्रपान छाड्नु नै वेश हुन्छ किनभने यसले फोक्सोलाई प्रभावित गरेर दम आक्रमणलाई निम्त्याउँछ।

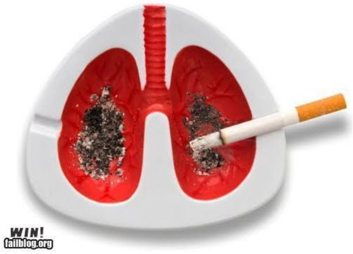

पेशागत संपर्क घटाउनुहोस्

पेशागत संपर्क कम गर्नुहोस् किनभने यसका कारणले फोक्सोमा धुवाँ र धुलो गएर दम नियन्त्रण गर्न गाह्रो हुन्छ।

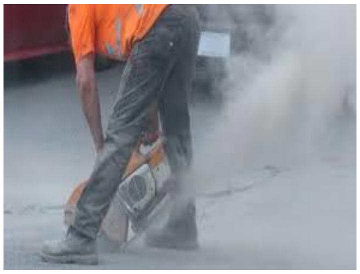

एलर्जीबाट बच्नुहोस्

धुँवा, धुलो, रसायन, फूल, रुख,

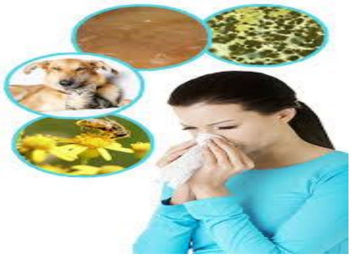

सिगरेट, जनवरहरु, आदिले दममा एलर्जी गराउउँदछ त्यसैले दमका विरामीहरुले यस्ता चिजहरुबाट बच्चनुपर्छ्

दमको उपचारमा प्रयोग हुने औषधी

दमको उपचार कसरी गरिन्छ?

दमको उपचार दुई तरिकाले गरिन्छ।

(१) दमको लक्षणहरु वा दम आक्रमणबाट छिटो राहत पाइने औषधीहरुबाट

(२) दमलाई पछि सम्मलाई नियन्त्रित पार्ने औषधीहरुबाट

दममा प्रयोग गरिने औषधीहरु

कोर्टिको स्टेरोइड: वायुमार्गमा सुजन कम गर्छ।

असरहरु:टाउको दुख्ने, आँखा

दुख्ने, धेरै पटक पिसाब लाग्ने, चक्कर लाग्ने, दिसामा रगत आउने

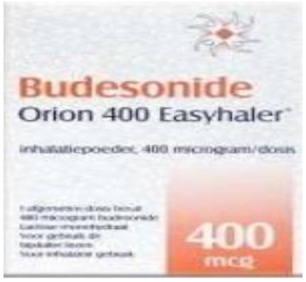

वा कालो रङको हुने, खुट्टाको

माथिल्लो भाग दुख्ने, वजन बढ्ने जस्ता समस्या हुन सक्छन्।

यस्ता असरहरु देखिएमा चिकित्सकलाई सम्पर्क गर्नुपर्छ।

कोर्टिको स्टेरोइड प्रयोग गर्दा यसको मात्रा (डोज) थप र घट चिकित्सकको परामर्श अनुसार गर्नु आवश्यक छ।

ब्रोन्कोडाईलेटर

फोक्सोको मांसपेशी लाई आराम दिन्छ।

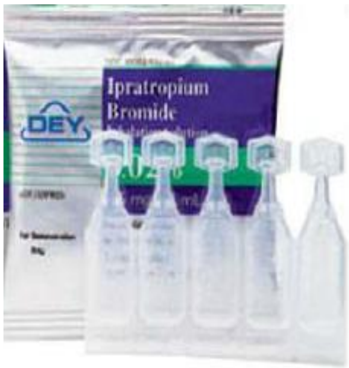

असरहरुः वान्ता आउँनु, चक्कर लाग्नु, मुख सुख्खा हुनु, धमिलो देख्नु, वजन घट्नु

यस्ता असरहरु देखिएमा चिकित्सकलाई सम्पर्क गर्नुपर्छ।

इन्हेलर

फोक्सोमा औषधी पुर्याउँने काम गर्दछ।

असरहरुः मुखको संक्रमण

तसर्थः प्रयोगपछि मुख कुल्ला गर्नुपर्छ।

नेबुलाईजर

बाफको मध्यमबाट औषधीफोक्सोमा पुर्याउँने काम गर्छ।

असरहरुः घाँटी दुख्नु, खोकी, नाकबाट रगत आउनु, पेट दुख्नु, पखाला लाग्नु,निन्द्रा नलाग्नु, मुखमा संक्रमण

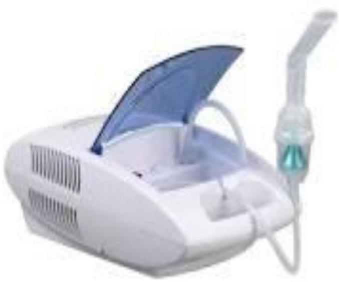

प्रयोगभन्दा पहिले २-३ सेकन्डसम्म साँस रोक्नुपर्छ। अनि त्यसपछि विस्तारै साँस बाहिर फाल्नुपर्छ।

थप जानकारीका लागिः
https://www.breathefree.com/breathingconditions/asthma(Accessed on 1st July, 2018)

सम्पर्कठेगानाः
anita.yadav2047@gmail.com

Ethical Approval from PURC

पोखरा विश्वविद्यालय
POKHARA UNIVERSITY
Kaski, Nepal

चलानी नं.:/Ref. No. 17-075-76

Thursday, August 09, 2018

Ms. Anita Yadav
Principal Investigator
School of Health and Allied Sciences
Faculty of Health Sciences
Pokhara University

Ref: **Ethical Approval of Research Proposal** entitled ***"Pharmacist Led Intervention on Inhalation Technique among Asthmatic patients for Improving Quality of Life in a Private Hospital of Nepal "***

Dear Ms. Anita Yadav

It is my pleasure to inform you that the above mentioned proposal has been approved by Pokhara University Research Center (PURC) and Institutional Review Committee (IRC).
As per IRC rules and regulations, the investigator has to strictly follow the protocol mentioned in the proposal. Any change in objective(s), problem statement, research questioner hypothesis methodology, implementation procedure, data management and budget that may be necessary in course of the implementation of the research proposal can only be made so and implemented after prior approval from this center. Thus, it is compulsory to submit the detail of such changes intended or desired with justification prior to actual change in the protocol.

Further, the researchers are directed to strictly abide by the IRC during the implementation of their research proposal and submit progress report and full copy of summary report upon completion.

If you have any questions, please contact the IRC Section at PURC.

Thank You.

GMKhan

Associate Professor Gulam Muhammad Khan
Executive Director Pokhara University Research Centre (PURC)
Pokhara Lekhnath-30, Kaski, Nepal.
gulamkhan@gmail.com / purc@pu.edu.np , http://pu.edu.np/university/purc/
+9841203704

Study Approval Letter

Crimson Hospital
Crimson College of Technology
(Pokhara University)

Tilottama-5, Manigram, Rupandehi, Nepal
Tel. : 00977-71-562744, 562745
Fax : 00977-71-562788
e-mail : hospital@cct.edu.np
Website : www.cct.edu.np

Ref. No. 532/074/075

Crimson Hospital
Crimson College of Technology
(Pokhara University)

11th July 2018

LETTER OF PERMISSION FOR STUDY APPROVAL

Dear Ms. Anita Yadav

M. Pharma (Clinical Pharmacy)

School of Health and Allied Science , Pokhara University.

Pokhara Lekhnath-30,Kaski,Nepal

The purpose of this letter is to grant you the permission to conduct the research titled **"Pharmacist Led Intervention on Inhalation Technique among Asthmatic Patients for Improving Quality of Life in a Private Hospital of Nepal"** for three months data collection at Crimson Hospital, Tillottama Municipality-05, Manigram, Rupandehi.

We expect from you as a researcher shall:

- maintain high standards of responsible research through intellectual honesty and integrity.
- be responsible for ensuring appropriate security for any confidential material held by you.
- respect the truth and the rights of those affected by your research;
- manage conflicts of interest so that ethical and scholarly considerations are not compromised,
- adopt appropriate methods to achieve the research aims;
- ensure the accuracy of your results and the work of assistants;
- conform to the policies of the hospital;
- follow other protocols as per direction of Hospital Director.

Wishing you all the best with the project.

For Crimson Hospital

....

Shekhar Prasad Pokhrel
Hospital Director

"गुणस्तरीय स्वास्थ्य सेवा सहज उपलब्धता, दक्ष जनशक्ति उत्पादन हाम्रो प्रतिबद्धता"
